# Effects of restrictive red blood cell transfusion on the prognoses of adult patients undergoing cardiac surgery: a meta-analysis of randomized controlled trials

**DOI:** 10.1186/s13054-018-2062-5

**Published:** 2018-05-31

**Authors:** Qi-Hong Chen, Hua-Ling Wang, Lei Liu, Jun Shao, Jiangqian Yu, Rui-Qiang Zheng

**Affiliations:** 1 Department of Critical Care Medicine, Northern Jiangsu People’s Hospital; Clinical Medical College, Yangzhou University, 98 Nantong West Road, Yangzhou, 225001 People’s Republic of China; 2 Department of Cardiology, Northern Jiangsu People’s Hospital; Clinical Medical College, Yangzhou University, 98 Nantong West Road, Yangzhou, 225001 People’s Republic of China

**Keywords:** Restrictive transfusion strategy, Liberal transfusion strategy, Cardiac surgery, Prognosis, Meta-analyses

## Abstract

**Purpose:**

Restrictive red blood cell transfusion strategies remain controversial in patients undergoing cardiac surgery. We performed a meta-analysis to assess the prognostic benefits of restrictive red blood cell transfusion strategies in patients undergoing cardiac surgery.

**Methods:**

We identified randomized clinical trials through the 9th of December 2017 that investigated a restrictive red blood cell transfusion strategy versus a liberal transfusion strategy in patients undergoing cardiac surgery. Individual patient data from each study were collected. Meta-analyses were performed for the primary and secondary outcomes. The risk of bias was assessed using the Cochrane Risk of Bias Tool. A trial sequential analysis (TSA)-adjusted random-effects model was used to pool the results from the included studies for the primary outcomes.

**Results:**

Seven trials involving a total of 8886 patients were included. The TSA evaluations suggested that this meta-analysis could draw firm negative results, and the data were sufficient. There was no evidence that the risk of 30-day mortality differed between the patients assigned to a restrictive blood cell transfusion strategy and a liberal transfusion strategy (odds ratio (OR) 0.98; 95% confidence interval (CI) 0.77 to 1.24; *p* = 0.87). Furthermore, the study suggested that the restrictive transfusion strategy was not associated with significant increases in pulmonary morbidity (OR 1.09; 95% CI 0.88 to 1.34; *p* = 0.44), postoperative infection (OR 1.11; 95% CI 0.95 to 1.3; *p* = 0.58), acute kidney injury (OR 1.03; 95% CI 0.92 to 1.14; *p* = 0.71), acute myocardial infarction (OR 1.01; 95% CI 0.80 to 1.27; *p* = 0.78), or cerebrovascular accidents (OR 0.97; 95% CI 0.72 to 1.30; *p* = 0.66).

**Conclusions:**

Our meta-analysis demonstrates that the restrictive red blood cell transfusion strategy was not inferior to the liberal strategy with respect to 30-day mortality, pulmonary morbidity, postoperative infection, cerebrovascular accidents, acute kidney injury, or acute myocardial infarction, and fewer red blood cells were transfused.

**Electronic supplementary material:**

The online version of this article (10.1186/s13054-018-2062-5) contains supplementary material, which is available to authorized users.

## Background

Anemia is common after cardiac surgery and is associated with significant increases in morbidity and mortality [1–3]. Red blood cell (RBC) transfusions can be lifesaving in patients with severe anemia and the purpose of perioperative RBC transfusion is to improve oxygen delivery in patients with anemia [4]. More than 50% of patients receive a postoperative transfusion, which uses a substantial proportion of blood supplies [5].

However, RBC transfusion has been associated with high rates of mortality and morbidity in critically ill patients [6]. It is associated with infection, acute lung injury, acute kidney injury, and death [7]. The infectious and non-infectious risks associated with transfusion support restrictive transfusion practices in several clinical settings [8]. Whether the restrictive approach to preoperative RBC transfusion in cardiac surgery safely achieves outcomes similar to those achieved by means of more liberal approaches remains unclear.

Recent studies have demonstrated that a restrictive strategy for RBC transfusion is not inferior to a liberal strategy with respect to death and other outcomes in patients undergoing cardiac surgery [9, 10]. The aim of this meta-analysis is to assess the effects of restrictive compared to liberal RBC transfusion on the prognoses of adult patients undergoing cardiac surgery.

## Methods

### Eligibility criteria

We included trials with the following features:

1. Types of studies: Randomized controlled clinical trials
2. Population: Patients undergoing cardiac surgery
3. Intervention: Patients receiving restrictive RBC transfusion
4. The following outcomes were included: a) primary outcome, 30-day mortality; b) secondary outcomes, pulmonary morbidity (including acute respiratory distress syndrome, acute lung injury, delayed extubation), postoperative infection (including deep sternal wound infection, leg wound infection, sepsis, etc.), cerebrovascular accident, acute kidney injury (including all stages, acute kidney injury requiring renal replacement treatment), and myocardial infarction.

### Search strategy and study selection

We searched the Medline, Elsevier, Embase, Cochrane (Central), Web of Science, and ClinicalTrials.gov databases from inception to December 9, 2017 for studies investigating the perioperative use of restrictive RBC transfusion in patients undergoing cardiac surgery. Two reviewers independently reviewed all abstracts and titles and excluded trials that were obviously irrelevant. The full texts of the articles were then reviewed independently in accordance with the inclusion and exclusion criteria. Any discrepancies were resolved by reaching a consensus regarding the inclusion or exclusion of a trial by discussion with a third reviewer.

### Data extraction and management

Two reviewers independently extracted the data using a standardized data extraction protocol. Any disagreements between the two reviewers were resolved by discussion. Information, including trial characteristics, included authors, year of publication, country of origin, study design, sample size, the inclusion and exclusion criteria, the methods of statistical adjustment, transfusion strategies, and study results, was extracted from the included studies.

### Trial sequential analysis

We conducted a trial sequential analysis (TSA) to prevent the risk of increases in random error by repeated updates according to the method we described previously [11]. A TSA-adjusted random-effects model was used to pool the results from the included studies for the primary outcomes. A two-sided TSA was performed to maintain a risk of 5% for type I error and a power of 80%. Additionally, an estimated function was used to calculate the required information size.

### Statistical analysis

Review Manager (version 5.3) was used for the meta-analysis. For each of the included studies, we calculated the odds ratio (OR) with 95% confidence intervals (CIs) for dichotomous outcomes. The heterogeneity among studies was calculated with the Mantel-Haenszel chi-square test and the I^2^ test. The statistical heterogeneity of the data was quantified. Obvious heterogeneity was defined as *p* < 0.05 using the Mantel-Haenszel chi-square test or an I^2^ > 50%. Furthermore, the funnel plot technique was used to assess the publication bias.

## Results

### Study location and selection

**Fig. 1:**
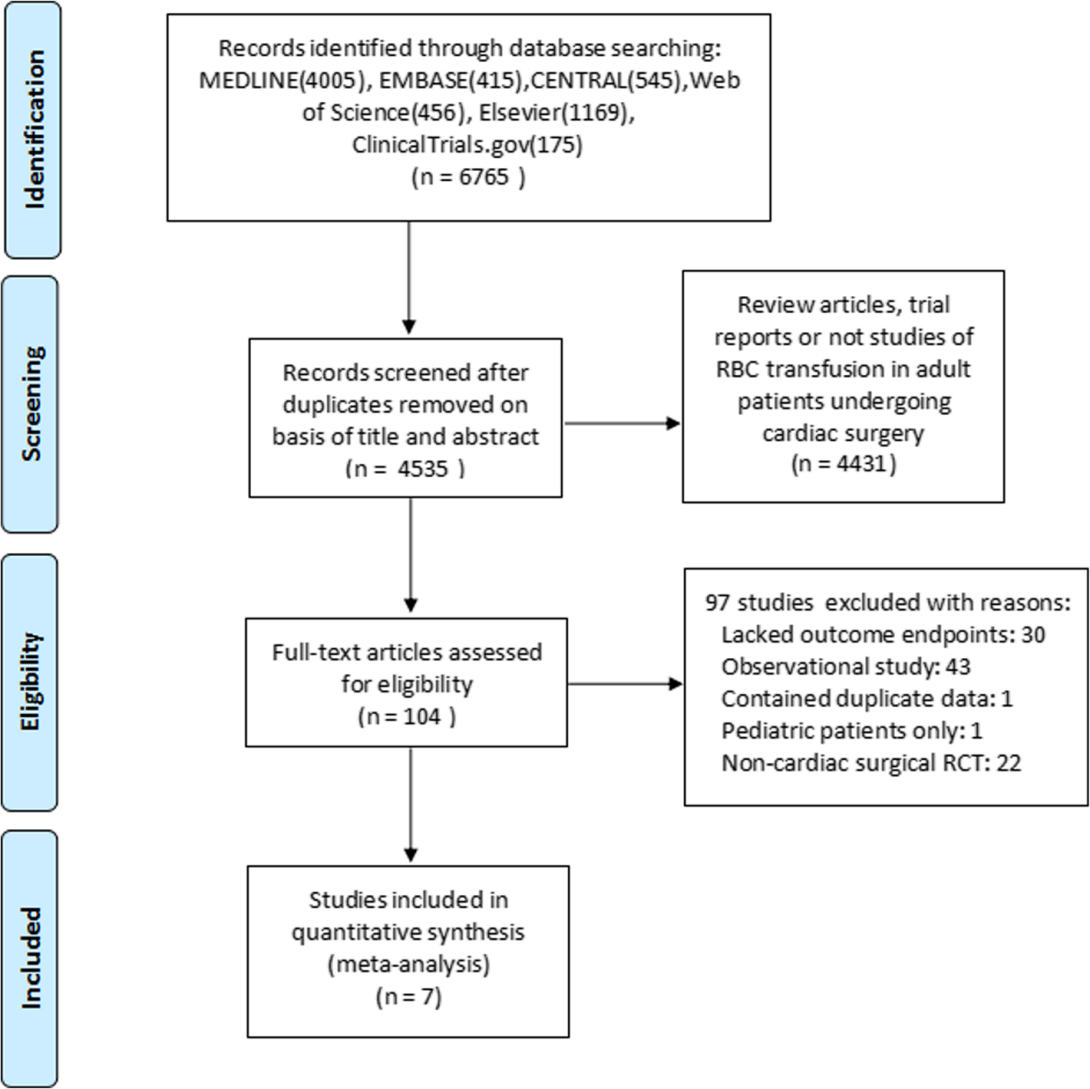
Flow diagram of the identified trials. *RCT* randomized controlled trial

Our search strategy identified a total of 6765 titles and abstracts. After screening the abstracts and title, 4535 publications were left after duplicates were removed. Among them, 4431 publications were non-relevant, which were therefore excluded. The remaining 104 publications were retrieved for an eligibility assessment; 97 publications were deemed ineligible and were therefore excluded. Seven studies with a total of 8886 patients were included in the final analysis [9, 10, 12–16] (Fig. 1).

### Characteristics of the trials

**Fig. 2:**
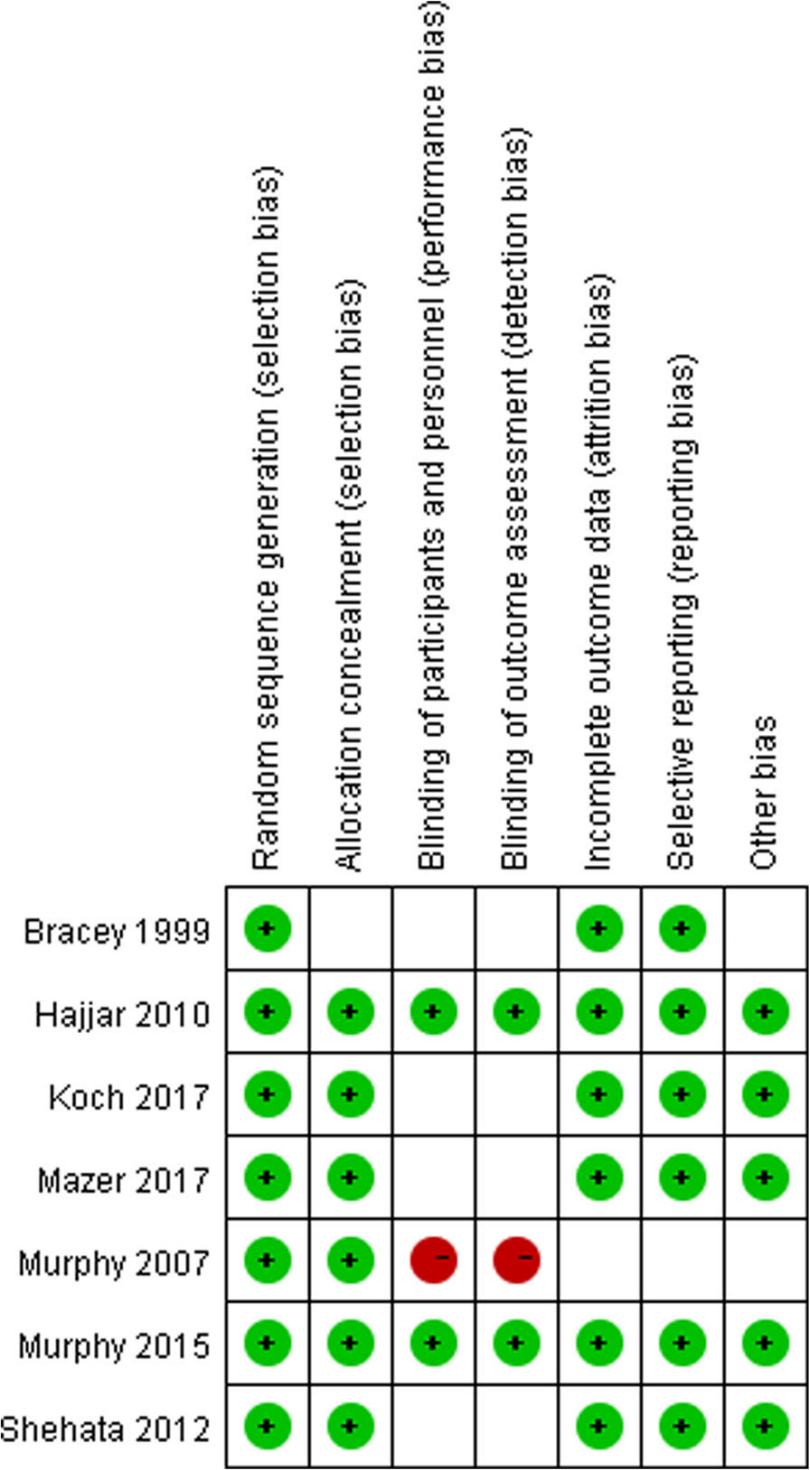
Risk of bias summary. Review of the authors’ judgements about each risk of bias item for each included study. *Red* indicates high risk, *green* indicates low risk, *blank* indicates unclear

We included seven trials that compared restrictive RBC transfusion with controls in patients undergoing cardiac surgery. The characteristics of the included trials are presented in Table 1. Four trials included only low-risk surgical patients who were undergoing elective cardiac surgery and excluded patients who were at the highest risk of requiring RBC transfusion [10, 12, 13, 15]. The other three trials included patients who were at the highest risk of requiring RBC transfusion [9, 14, 16]. Patients allocated to the restrictive RBC transfusion group were infused with fewer RBCs compared to patients in the liberal-threshold group. The median number of cell salvage and allogeneic RBC units transfused per patient ranged from one to three in the four studies [9, 12–14]. RBC transfusion rates reported in three trials ranged from 44 to 75% [10, 13, 15]. The other trial did not report the units of RBC transfusion or transfusion rate [7]. The results of random sequence generation are shown in Fig. 2.

**Table 1:** Characteristics of included studies

Study	N	Age			Strategy of blood transfusion				Units of RBC transfusion or transfusion rate	
		Restrictive	Control	Setting	Restrictive		Control		Restrictive	Control
					Triggered Hb	Observed Hb	Triggered Hb	Observed Hb		
Bracey et al.1999 [12]	428	61 ± 11	62 ± 11	Elective CABG	Hb < 8 g/dl	9.1 g/dl	Hb < 9 g/dl	9.7 g/dl	0.9 ± 1.5	1.4 ± 1.8
Murphy et al. 2007 [16]	321	NS	NS	Elective or urgent cardiac surgery	Hb < 7 g/dl	NS	Hb < 8 g/dl	NS	NS	NS
Hajjar et al. 2010 [13]	502	58.6 ± 12.5	60.7 ± 12.5	Elective cardiac surgery	Hct < 24%	9.6 g/dl	Hct < 30%	10.7 g/dl	0 (0–2)	2 (1–3)
Shehata et al. 2012 [15]	50	67.2 ± 11.2	68.8 ± 9.2	Cardiac surgery with CARE score of 3 or 4	Hb < 7.5 g/dl	9.1 g/dl	Hb < 10 g/dl	10.7 g/dl	11 (44)	17 (68)
Murphy et al. 2015 [14]	2003	69.9(63.1 –76.0)	70.8(64.1–76.7)	Elective or urgent cardiac surgery	Hb < 7.5 g/dl	9.0 g/dl	Hb < 9 g/dl	9.8 g/dl	1 (0–2)	2 (1–3)
Koch et al. 2017 [10]	717	59 ± 15	60 ± 13	Elective CABG or HVR	Hct < 24%	28%	Hct < 28%	30%	195 (54)	265 (75)
Mazer et al. 2017 [9]	4860	72 ± 10	72 ± 10	Cardiac surgery with a EuroSCORE I of 6 or more	Hb < 7.5 g/dl	Hb < 8.5 g/dl	Hb < 9.5 g/dl	10.5 g/dl?	2 (1–4)	3 (2–5)

*CABG* coronary artery bypass grafting, *Hb* hemoglobin, *Hct* hemotocrit, *HVR* heart valve replacement, *NS* normal saline

### Trial sequential analysis

**Fig. 3:**
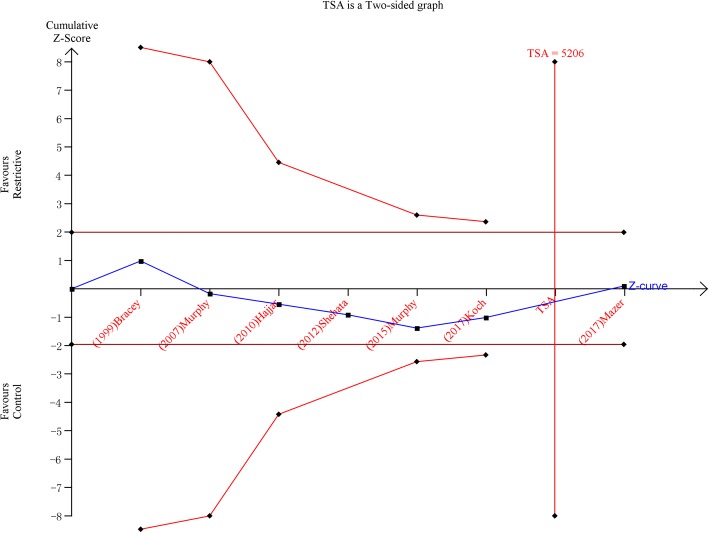
Trial sequential analysis for mortality in the randomized controlled trials with a two-sided boundary and an incidence of 2.78% in the control arm and an incidence of 1.42% in the treatment arm

A TSA sensitivity analysis including all trials revealed that the diversity-adjusted information size was 8886 patients. The cumulative z-curve did not cross the conventional boundary for benefit or the trial sequential monitoring boundary for benefit but did cross the estimated information size boundary (Fig. 3). The TSA evaluations suggested that this meta-analysis could draw firm negative results, and the data were sufficient.

### Mortality

**Fig. 4:**
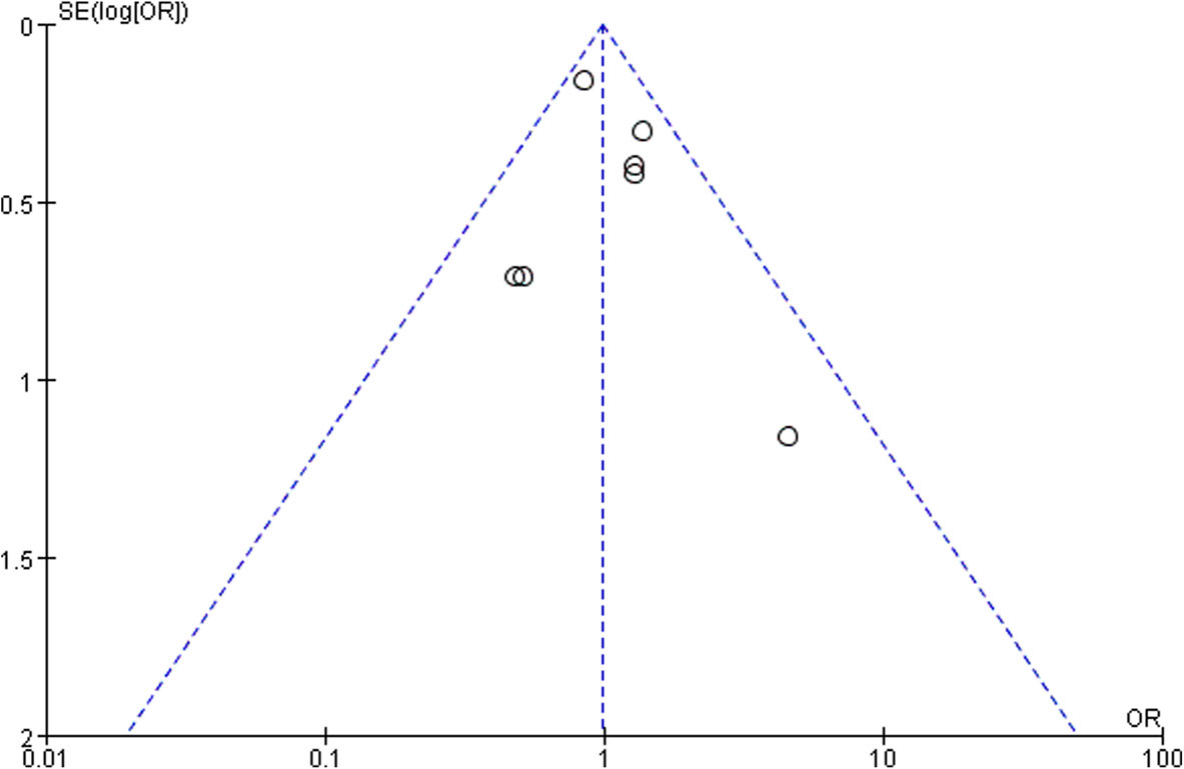
Funnel plot of the mortality demonstrating that no publication bias existed

**Fig. 5:**
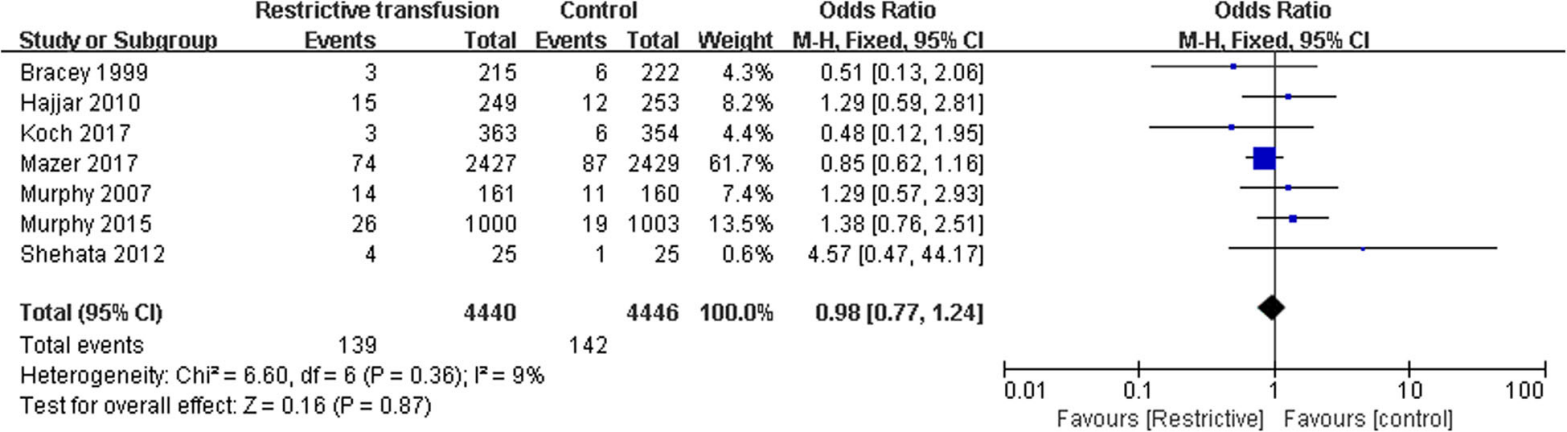
Effect of restrictive red blood cell transfusion on postoperative mortality in adult patients undergoing cardiac surgery: a meta-analysis of randomized controlled trials

The effect of restrictive RBC transfusion on 30-day mortality rates was estimated from seven trials that included a total of 8886 patients. A total of 139 deaths occurred among 4440 patients who were allocated to the restrictive RBC transfusion group compared with 142 deaths among the 4446 patients allocated to the control group. No evidence of publication bias was detected after a funnel plot analysis (Fig. 4), and the heterogeneity was determined to be non-significant (*p* = 0.36, I^2^ = 9). There was no evidence that the risk of 30-day mortality differed between the patients assigned to the restrictive RBC transfusion and control groups (OR 0.98; 95% CI 0.77 to 1.24; *p* = 0.87; Fig. 5).

### Secondary outcomes

Five studies (3658) reported pulmonary morbidity as an outcome. The results revealed that there was no significant reduction in the risk of pulmonary morbidity with restrictive RBC transfusion (*p* = 0.42, Table 2). Furthermore, the study suggested that the restrictive transfusion strategy was not associated with significant increases in pulmonary morbidity, postoperative infection, acute kidney injury, acute myocardial infarction, or cerebrovascular accidents (Table 2, Additional files 1, 2, 3, 4, and 5).

**Table 2:** Effects of red blood cell transfusion by outcome

	Number of studies	Number of patients	Fixed effects Odds ratio (95% CI)	Fixed effects *p* value	I^2^ (%)	Heterogeneity *p* value
Mortality	7	8886	0.98 (0.77–1.24)	0.87	9	0.36
Pulmonary morbidity	5	3658	1.09 (0.88–1.34)	0.42	0	0.44
AKI	6	8355	1.03 (0.92–1.14)	0.65	0	0.71
AMI	4	7302	1.01 (0.80–1.27)	0.95	0	0.78
Infectious morbidity	6	8444	1.11 (0.95–1.3)	0.19	0	0.58
Cerebrovascular accident	6	8528	0.97 (0.72–1.30)	0.84	0	0.66

*AKI* acute kidney injury, *AMI* acute myocardial infarction

## Discussion

Restrictive RBC transfusion strategies remain controversial in patients undergoing cardiac surgery [3, 7]. Thus, the effect of restrictive versus liberal transfusion strategies on clinical outcomes in patients undergoing cardiac surgery remains to be defined. Our meta-analysis demonstrated that the OR for 30-day mortality did not favor a restrictive transfusion strategy or a liberal transfusion strategy in randomized controlled trials of adult patients undergoing cardiac surgery. Furthermore, a restrictive RBC transfusion strategy was not inferior to a liberal strategy with respect to pulmonary morbidity, postoperative infection, cerebrovascular accident, acute kidney injury, or acute myocardial infarction, and fewer RBCs were transfused.

Some studies have suggested that the transfusion of RBCs is associated with many harmful effects, such as infection, acute lung injury, acute kidney injury, prolonged hospital stays, and increased mortality and hospital costs [7, 17]. A restrictive threshold for transfusion is likely to be favored because it requires the use of fewer units of RBCs [18, 19]. Considering the known risks of RBC transfusions and the observational studies linking transfusion with increased adverse complications [20], clinicians have been adopting restrictive RBC transfusion strategies in cardiac surgery [21]. However, restrictive RBC transfusion strategies remain controversial in patients undergoing cardiac surgery [22]. Patients undergoing cardiac surgery have a lower cardiovascular reserve and restrictive RBC transfusion may increase the risk of anemia-induced tissue hypoxia [23]. Our meta-analysis provides evidence that restrictive transfusion is not associated with the risk of adverse outcomes such as infection, acute kidney injury, and pulmonary morbidity. However, the definitions of those secondary outcomes differed between studies. For instance, the KDIGO criteria were adopted to diagnose acute kidney injury in TRICS 3 trial [9], but Hajjar et al. applied the RIFLE classification [13], and some others employed dialysis-dependent or 50% or greater increase in serum creatinine [15, 16]. Nonetheless, this meta-analysis suggests that restrictive transfusion strategies are as safe as liberal strategies in patients undergoing cardiac surgery.

Observational studies of adult patients undergoing cardiac surgery have shown strong associations between RBC transfusion and high mortality [24, 25]. In the Transfusion Indication Threshold Reduction (TITRe2) clinical trial, 90-day mortality was higher with restrictive postoperative RBC transfusion than with a liberal threshold [14]. A meta-analysis of there randomized controlled trails reported that the odds for mortality favored a liberal RBC transfusion strategy rather than a restrictive RBC transfusion strategy, but the difference between strategies was not statistically significant [20]. However, the recently published TITRe3 trial did not provide evidence supporting this. The study showed that in patients undergoing cardiac surgery who were at moderate to high risk for death, a restrictive RBC transfusion strategy was noninferior to a liberal strategy with respect to the composite outcome of death from any cause [9]. Similar to the TRICS 3 trial, our meta-analysis demonstrated that a restrictive RBC transfusion strategy is not inferior to a liberal strategy with respect to 30-day mortality. To avoid the risk of random error increase due to repeated updates, a sensitivity analysis of the TSA was performed. The TSA evaluations suggested that this meta-analysis could draw firm negative results, and the data were sufficient. Thus, the restrictive RBC transfusion strategy was not inferior to the liberal strategy with respect to 30-day mortality.

There are some procedures and techniques to reduce RBC transfusion in patients undergoing cardiac surgery [26]. In 2010, the World Health Organization encouraged all member countries to implement patient blood management (PBM) programs employing multiple combined strategies to increase and preserve autologous erythrocyte volume to restrict RBC transfusions [27]. PBM programs included preoperative optimization of hemoglobin levels, blood-sparing techniques, and standardization of transfusion practice [28, 29]. Since then the PBM program has been adopted to minimize blood loss in patients undergoing cardiac surgeries [30]. Gross et al. [31] reported that implementing meticulous surgical techniques, a goal-directed coagulation algorithm, and a more restrictive transfusion threshold in combination resulted in an obvious decrease in RBC transfusions and lower total direct costs. Despite the benefits of PBM, many barriers limit translation of PBM guidelines into clinical practice worldwide, particularly in the absence of interdisciplinary commitment, lack of resources, and general concerns. Strategies for overcoming the obstacles include the use of bundles of care and specifically designed measures on the basis of local conditions [32].

Several pharmacologic agents have been used to decrease intraoperative blood loss, which is helpful to reduce RBC transfusion. Antifbrinolytic agents, including tranexamic acid and epsilon aminocaproic acid, have been extensively studied, and they decrease hemostatic activation, reduce bleeding, and decrease allogeneic RBC transfusions [33, 34]. Furthermore hemostatic treatment with fibrinogen concentrate in patients undergoing aortic surgery significantly reduced allogeneic blood transfusion [35]. In addition, several erythropoietin dosing regimens and duration treatment increase red cell mass and reduce allogeneic blood transfusions [36]. Erythropoietin administered before cardiac surgery seems effective in reducing the need for RBCs without increasing adverse events, hence reducing transfusion requirements [37, 38]; however, it is still controversial [39]. Recently Urena et al. [40] showed that combined erythropoietin and iron therapy failed to reduce RBC transfusion in anemic patients undergoing cardiac surgery.

This meta-analysis has several limitations. First, the hemoglobin thresholds of the restrictive RBC transfusion strategies varied between the trials. Thus, the appropriate threshold remains to be defined and could vary for different patients. Second, the types of cardiac surgery differed among the included studies and patients undergoing different types of cardiac surgery may have different tolerances to restrictive transfusion strategies.

## Conclusions

The available evidence from our updated meta-analysis suggests that the OR for 30-day mortality did not favor a restrictive or liberal transfusion strategy in randomized controlled trials of adult patients undergoing cardiac surgery. Our meta-analysis is the best available evidence that restrictive RBC transfusion is as effective and safe as liberal transfusion strategies in adult cardiac surgery, although the appropriate threshold remains to be defined and could vary for different patients.

## Additional files

Additional file 1: Effect of restrictive red blood cell transfusion on pulmonary morbidity. Forest plot of adult patients undergoing cardiac surgery. Pulmonary morbidity includes acute respiratory distress syndrome, acute lung injury, delayed extubation. ARDS and ALI are according to the Berlin definition. Delayed extubation defined by inability to extubate the patients within 24 h after the completion of the surgical procedure. (PNG 5 kb)

Additional file 2: Effect of restrictive red blood cell transfusion on postoperative acute kidney injury (*AKI*). Forest plot of adult patients undergoing cardiac surgery. AKI is defined according to the KDIGO or RIFLE criteria or as dialysis-dependent or 50% or greater increase in serum creatinine. (PNG 6 kb)

Additional file 3: Effect of restrictive red blood cell transfusion on postoperative infections. Forest plot in adult patients undergoing cardiac surgery. Pneumonia was defined as autopsy diagnosis or roentgenographic infiltrate and at least two of the following three criteria: fever, leukocytosis, and positive sputum culture; or deep sternal or leg wound infection requiring intravenous antibiotics and/or surgical debridement. (PNG 6 kb)

Additional file 4: Effect of restrictive red blood cell transfusion on postoperative acute myocardial infarction (*AMI*). Forest plot of adult patients undergoing cardiac surgery. Myocardial infarction was defined according to the task force for the European Society of Cardiology, the American College of Cardiology Foundation, the American Heart Association, and the World Heart Federation. (PNG 5 kb)

Additional file 5: Effect of restrictive red blood cell transfusion on postoperative cerebrovascular accident. Forest plot of adult patients undergoing cardiac surgery. Cerebrovascular accident is defined as new focal neurological deficit lasting more than 24 h confirmed by clinical assessment and brain imaging. (PNG 6 kb)

## Abbreviations

AKI: Acute kidney injury
AMI: Acute myocardial infarction
CABG: Coronary artery bypass grafting
CARE: Cardiac anesthesia risk score
EuroSCORE: European System for Cardiac Operative Risk Evaluation
Hb: Hemoglobin
HVR: Heart valve replacement
ICU: Intensive care unit
RBC: Red blood cell
TSA: Trial sequential analysis
