# Supplementary figures and images for: Effects of restrictive red blood cell transfusion on the prognoses of adult patients undergoing cardiac surgery: a meta-analysis of randomized controlled trials
Source: Crit Care. 2018 May 31;22:142. doi: 10.1186/s13054-018-2062-5 (PMC5977455; doi:10.1186/s13054-018-2062-5)

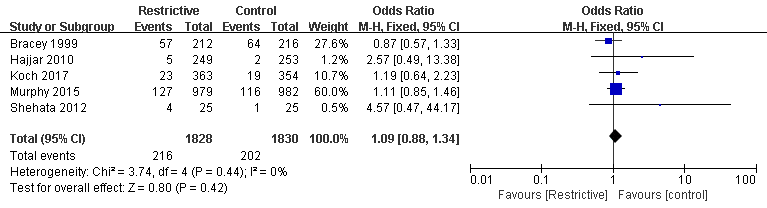

Supplement: Supplementary file 1 — Effect of restrictive red blood cell transfusion on pulmonary morbidity. Forest plot of adult patients undergoing cardiac surgery. Pulmonary morbidity includes acute respiratory distress syndrome, acute lung injury, delayed extubation. ARDS and ALI are according to the Berlin definition. Delayed extubation defined by inability to extubate the patients within 24 h after the completion of the surgical procedure. (PNG 5 kb) [file 13054_2018_2062_MOESM1_ESM.png]

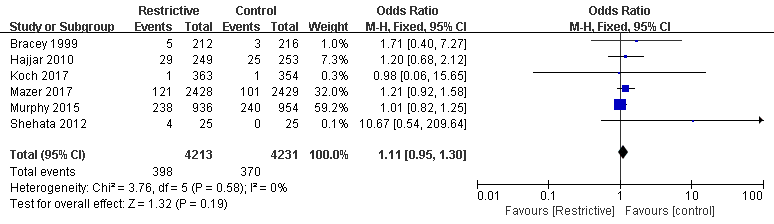

Supplement: Supplementary file 2 — Effect of restrictive red blood cell transfusion on postoperative acute kidney injury (AKI). Forest plot of adult patients undergoing cardiac surgery. AKI is defined according to the KDIGO or RIFLE criteria or as dialysis-dependent or 50% or greater increase in serum creatinine. (PNG 6 kb) [file 13054_2018_2062_MOESM2_ESM.png]

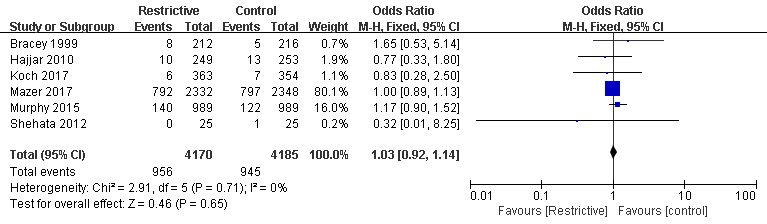

Supplement: Supplementary file 3 — Effect of restrictive red blood cell transfusion on postoperative infections. Forest plot in adult patients undergoing cardiac surgery. Pneumonia was defined as autopsy diagnosis or roentgenographic infiltrate and at least two of the following three criteria: fever, leukocytosis, and positive sputum culture; or deep sternal or leg wound infection requiring intravenous antibiotics and/or surgical debridement. (PNG 6 kb) [file 13054_2018_2062_MOESM3_ESM.png]

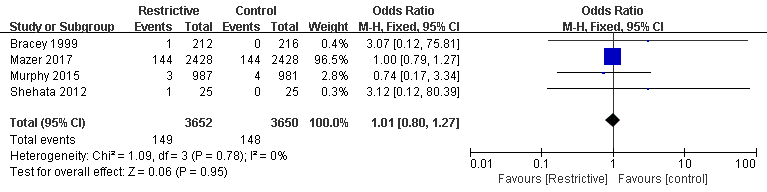

Supplement: Supplementary file 4 — Effect of restrictive red blood cell transfusion on postoperative acute myocardial infarction (AMI). Forest plot of adult patients undergoing cardiac surgery. Myocardial infarction was defined according to the task force for the European Society of Cardiology, the American College of Cardiology Foundation, the American Heart Association, and the World Heart Federation. (PNG 5 kb) [file 13054_2018_2062_MOESM4_ESM.png]

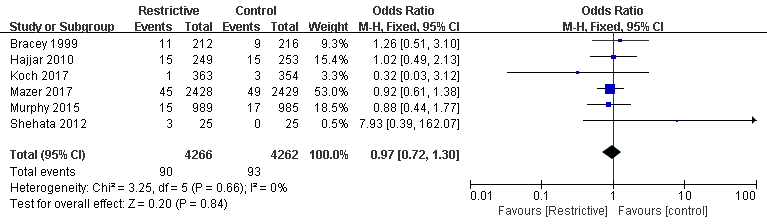

Supplement: Supplementary file 5 — Effect of restrictive red blood cell transfusion on postoperative cerebrovascular accident. Forest plot of adult patients undergoing cardiac surgery. Cerebrovascular accident is defined as new focal neurological deficit lasting more than 24 h confirmed by clinical assessment and brain imaging. (PNG 6 kb) [file 13054_2018_2062_MOESM5_ESM.png]
